# Supplementary material for: High-Sensitive Spatial Proteomics for Pancreatic Cancer Progression Analysis
Source: bioRxiv. 2025 May 5:2025.05.01.651678. Preprint. [Version 1] doi: 10.1101/2025.05.01.651678 (PMC12247709; doi:10.1101/2025.05.01.651678)
Supplement: Supplement 1 [file NIHPP2025.05.01.651678v1-supplement-1.pdf]

bioRxiv preprint doi: https://doi.org/10.1101/2025.05.01.651678; this version posted May 5, 2025. The copyright holder for this preprint (which was not certified by peer review) is the author/funder, who has granted bioRxiv a license to display the preprint in perpetuity. It is made available under aCC-BY 4.0 International license.

Supplementary Information

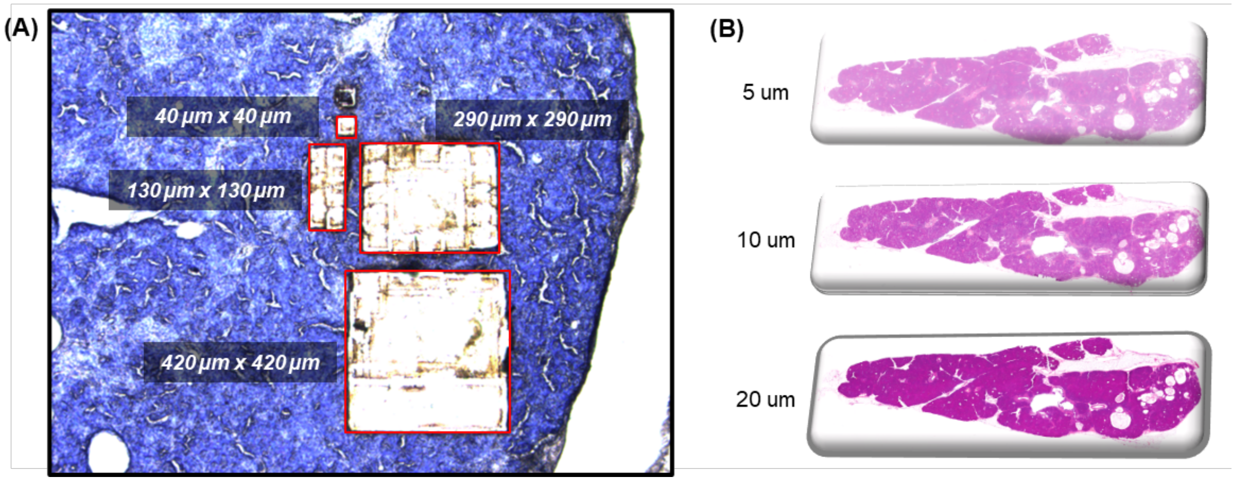

Figure S1. (A) Laser microdissection (LMD) capture areas shown for varying cell counts on a 5 µm thick pancreas tissue section. (B) H&E-stained images of pancreas tissue sections prepared at three different

bioRxiv preprint doi: https://doi.org/10.1101/2025.05.01.651678; this version posted May 5, 2025. The copyright holder for this preprint (which was not certified by peer review) is the author/funder, who has granted bioRxiv a license to display the preprint in perpetuity. It is made available under aCC-BY 4.0 International license.

thicknesses: 5 µm, 10 µm, and 20 µm, demonstrating the structural consistency in tissue morphology with slide thickness.

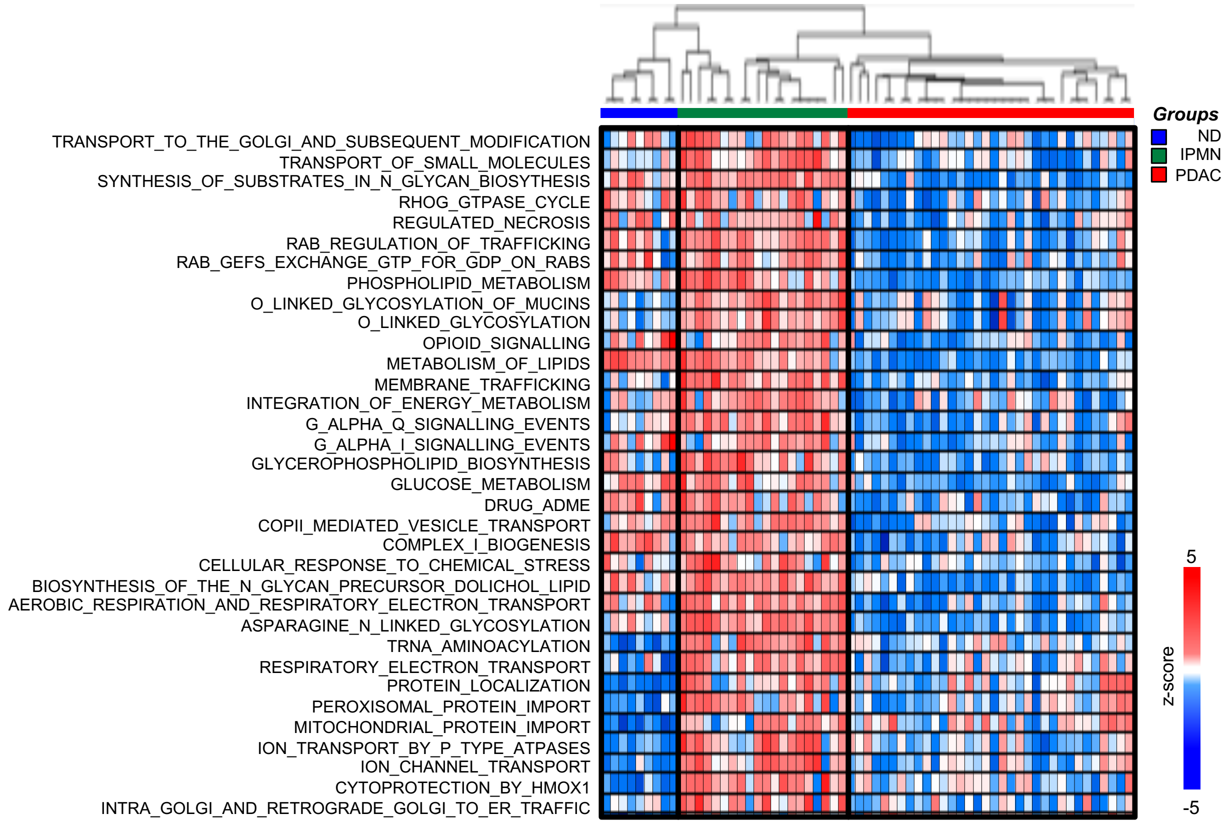

Figure S2. Heatmap illustrating Reactome pathway activation scores from ssGSEA for pathways upregulated in IPMN comparing to normal ducts but downregulated in PDAC. Rows represent individual Reactome pathways, while columns correspond to individual samples grouped by disease condition (ND in blue, IPMN in green, and PDAC in red). The color scale indicates z-scores, with red representing higher activation and blue representing lower activation.
